# A Review of Practice-Changing Therapies in Oncology in the Era of Personalized Medicine

**DOI:** 10.3390/curroncol31040143

**Published:** 2024-04-02

**Authors:** Mariana Pilon Capella, Khashayar Esfahani

**Affiliations:** 1 Lady Davis Institute and Segal Cancer Centre, Jewish General Hospital, Departments of Medicine and Oncology, McGill University, Montreal, QC H3T 1E9, Canada; mpilonc@gmail.com; 2 St Mary’s Hospital, Departments of Medicine and Oncology, McGill University, Montreal, QC H3T 1M5, Canada

**Keywords:** precision oncology, CAR-T cell, BiTE, antibody-drug conjugates, microbiome, immunotherapy, tumor-agnostic therapies

## Abstract

In the past decade, a lot of insight was gathered into the composition of the host and tumor factors that promote oncogenesis and treatment resistance. This in turn has led to the ingenious design of multiple new classes of drugs, which have now become the new standards of care in cancer therapy. These include novel antibody-drug conjugates, chimeric antigen receptor T cell therapies (CAR-T), and bispecific T cell engagers (BitTE). Certain host factors, such as the microbiome composition, are also emerging not only as biomarkers for the response and toxicity to anti-cancer therapies but also as potentially useful tools to modulate anti-tumor responses. The field is slowly moving away from one-size-fits-all treatment options to personalized treatments tailored to the host and tumor. This commentary aims to cover the basic concepts associated with these emerging therapies and the promises and challenges to fight cancer.

## 1. From the Past to the Present

Cancer is a disease as old as humanity itself, with the earliest known descriptions of it appearing in several papyri from ancient Egypt. From these early descriptions, the disease was feared and appreciated as a systemic problem, one which would need to be uprooted by any means possible. This led over the centuries to ever-increasing radical treatment strategies, such as Halstead’s disfiguring extensive mastectomy or near-lethal non-target specific chemotherapy regimens requiring salvage bone marrow transplants. It was not until the last two decades that both medical and surgical studies demonstrated that less was more and that a more restrained treatment strategy could lead to the same efficacy with much less morbidity [1]. With this paradigm shift, the foundation for the emergence of precision oncology and personalized medicine was laid. This was subsequently mainstreamed by large-scale efforts to better understand the composition and behavior of the cancer cells, such as The Cancer Genome Atlas (TCGA), which molecularly characterized over 20,000 primary tumors and matched normal samples spanning 33 cancer types [2]. A roadmap to success was finally laid, and all we needed was the technologies to target the newly discovered pathways that tumors use to their advantage. In this paper, we will discuss a few of these promising approaches, including novel drug delivery mechanisms in the form of cleverly designed antibodies, chimeric antigen receptor T-cell therapy (CAR-T), the emerging field of microbiome manipulation, and tumor-agnostic treatment approaches. These are summarized in Table 1.

## 2. Tumor-Agnostic Drug Indications: A New Perspective

Historically, novel drugs have been approved for a specific tumor type. With our better understanding of the underlying genetics of the tumor, we have seen that certain druggable mutations are not tumor-specific, i.e., they can occur across all tumor types with a varying degree of incidence. One such breakthrough was the discovery of the mismatch-repair (MMR) deficiency system. The DNA MMR, a set of various intracellular enzymes that exist extensively in organisms ranging from prokaryotes to eukaryotes, is a highly conserved repair mechanism in cellular evolution. In essence, the MMR system is the cell’s mechanism for proofreading and correcting errors in the genetic code that occur during DNA replication secondary to damage caused by external physical or chemical insults. The MMR system comprises a series of specific DNA mismatch repair enzymes, such as mutL homologue 1 (MLH1), postmeiotic segregation increased 2 (PMS2), mutS homologue 2 (MSH2), and mutS 6 (MSH6). Mutations or deficiencies in one or many of these genes can lead to a compromised MMR system, contributing to genomic instability with a less proficient repair of DNA in the tumor cells, an accumulation of genetic mutations, and an ultimate result of overly aberrant neoantigen expression. With the increased neoantigen load, the tumors become more visible to the immune system, thus becoming a prime target for immunotherapy drugs.

Immunotherapy, more specifically checkpoint inhibitors, has revolutionized the field of oncology and has become a pillar in cancer care. We previously reviewed the topic in another review article [3]. The success of immunotherapy is often very unpredictable and varies a lot between different patients and tumor types. However, one prognostic and predictive factor that was consistently shown to be associated with better responses and survival was the MMR deficiency status of a tumor. Taking colorectal cancer as an example, this tumor rarely responds to immunotherapy. In tumors with MMR deficiency, up to 40% of responses are observed, with many of them leading to cures [4]. In another study, pembrolizumab was shown to induce similar drastic responses across different MMR-deficient tumor types [5]. This, in turn, led to the first-ever tumor-agnostic approval of a cancer drug (pembrolizumab) by the Food and Drug Administration (FDA) in 2016, followed by Nivolumab in 2017. Other drugs are likely to follow suit, as the paradigm is shifting from treating tumors based on their cellular origin to focusing on their genetic composition, which is not restricted to any particular type of tumor. 

## 3. Novel Drug Delivery Mechanisms: Engineered Antibodies

The National Cancer Institute’s monumental drug screening program in the late 20th century for identifying novel chemotherapeutic agents occurred between the discovery of the first chemotherapies during World War II and the time period when most of today’s chemotherapy drugs were developed [6]. Rather than searching for new chemotherapies, the research of the past two decades has focused on designing novel delivery mechanisms for old drugs to maximize the payload to the tumor while at the same time minimizing off-target, undesirable side effects. One such mechanism has been the advent of antibody-drug conjugates (ADCs). With this novel design, chemotherapy is now conjugated (i.e., attached) to an antibody directed at a surface tumor antigen, allowing for the delivery of extremely high levels of chemotherapy directly to the tumor bed. As an example, the classic treatment of metastatic HER2+ breast cancer consists of the separate injection of anti-HER2 antibodies and intravenous chemotherapy. The ADC trastuzumab-deruxtecan, trastuzumab being the antibody against HER2 and deruxtecan the conjugated topoisomerase I inhibitor, allows for a significantly higher delivery of chemotherapy to the tumor and showed remarkable efficacy in heavily pretreated patients, with a remarkable 60% response rate [7]. This drug was then shown to be superior to the standard-of-care second-line therapy, and studies are currently underway to assess its efficacy in first-line therapy [8]. We have also seen significant responses in patients with lower HER2 expression, which classically do not qualify as HER2-positive disease [9]. This has led to a complete reclassification of how we look at HER2 positivity in tumors, with a new category labeled as HER2-low disease. Other major landmark studies with antibody drug-conjugates are underway in other solid tumors [8,10].

Another modern feat of drug engineering has been the bispecific T-cell engager (BiTE) therapies. These drugs can be described as biological matchmakers, physically linking cancer cells directly to immune cells. BiTE technology is thus designed to overcome cancer cells’ evasion of the immune system by engaging patients’ own T cells to directly bind to the cancer cells. These drugs first showed remarkable progress in hematological malignancies [11]. Lately, we have also seen their emergence in solid tumors. As an example, small-cell lung cancer remains a lethal disease, with few options beyond first-line therapy in the metastatic setting. The BiTE drug tarlatamab, engaging the delta-like ligand 3 on the surface of the tumor to the CD3 receptor of T cells, has shown a breakthrough efficacy signal, with objective response rates in over 40% of patients [12]. Other studies looking at novel BiTE therapies are currently underway across numerous tumor types.

## 4. Genetically Engineered Cell Therapies

Tumors express antigens that differentiate them from normal healthy tissues. These tumor-associated antigens are not only a diagnostic tool, such as the carcinoembryonic antigen (CEA) used in the monitoring of colon cancer, but can also be a target for treatment. Over the past decades, various strategies have been used to target these abnormally expressed antigens. These included vaccines and adoptive cell transfer strategies, amongst others. Undoubtedly, the most successful strategy has been the development of chimeric antigen receptor T-cell therapy (CAR-T). CARs are engineered synthetic receptors that function to redirect lab-engineered lymphocytes, most commonly T cells, to recognize and eliminate cells expressing a specific target antigen. CAR-T cells are generated by removing T cells from a patient’s blood, engineering the cells in the lab to express the CAR recognizing the tumor antigen of interest, and then reinfusing those cells back into the patient.

CAR-T therapies work best in cancers that uniformly express an antigen of interest. It is for this reason that this treatment strategy has had outstanding success in hematological malignancies. Most notably, in B cell acute lymphoblastic leukemia (B-ALL), up to a 90% complete remission rate with a CAR-T targeting the CD19-20 antigens has been observed in previously treatment-refractory cases [13]. There are now six FDA-approved CAR-T therapies for leukemias, lymphomas, and myeloma, each representing a breakthrough strategy and a novel pillar of cancer therapy in these indications [14,15,16,17,18].

CAR-T cell therapy’s compelling success in treating hematologic malignancies paved its development in solid tumors [19,20,21]. However, this strategy for solid tumors has been much less encouraging, with mitigated efficacy signals and potentially significant toxicity [13,19]. Unlike hematological malignancies where the antigen of interest is uniformly expressed and maintained over time in all cancer cells, in solid tumors, antigen expression is widely heterogeneous and variable over time. Furthermore, in certain cases where a suitable antigen is found and a CAR-T is designed, other factors such as the solid tumor microenvironment impede the success of this strategy [22]. Unlike hematological malignancies where the tumor cells are free-floating in the blood or in the lymph nodes, solid tumors tend to create a shield around them in the form of a dense mesothelial barrier that impedes the penetrance of immune cells, whether native or chimeric. Identifying tumor-specific antigens and refining CAR construct designs to improve T-cell trafficking and overcome the hostile tumor microenvironment are two primary areas of current research aimed at developing effective CAR-T-cell therapies for solid tumors [23]. Studies are looking at leveraging the power of CAR-T in combination with other strategies, such as chemotherapy, radiation therapy, or targeted treatments, to overcome the resistance barriers to their penetrance in the tumor bed [24].

## 5. The Microbiome: A Weaponizable Tool against Cancer 

The microbiome refers to a diverse community of microorganisms that inhabit many parts of the human body, including the gastrointestinal tract, skin, and mucosal surfaces. In symbiosis with the host, the microbial communities maintain homeostasis and regulate immune function. However, microbiota dysbiosis can lead to an imbalance in immune responses, potentially contributing to the development of inflammatory conditions and diseases, including cardiovascular diseases, respiratory diseases, autoimmune diseases, and malignancies. It is believed that over 70% of our lymphocytes transit through the gut at some point during their lifetime and get modulated by the gut microbiota [25]. These interactions shape the immune responses that happen through our body, either for the better (such as in the case of enhanced cancer surveillance) or for the worse (such as in inflammatory autoimmune conditions). Numerous studies have linked factors modulating the host’s microbiota, such as extremes of body weight, dietary composition, and the use of antibiotics and prebiotics, with different immune outcomes and disease associations [26,27,28].

Initially, it was discovered that the treatment of mice with antibiotics would result in the reduced efficacy of numerous anticancer drugs, such as platinums, cyclophosphamide, gemcitabine, and fluoropyridines, that are to this date used across numerous cancer indications [27]. Many mechanisms were later established, including suppression of programmed cell death, epigenetic changes, altered gene amplification, and DNA break repair, that can lead to anticancer drug inactivation. Albeit very intriguing, these findings have not led to therapeutic interventions beyond an increased awareness of the harm of antibiotic use and better antibiotic stewardship guidelines.

The real breakthrough in the field of microbiome studies has come in the era of immunotherapy, more specifically with the advent of the immune checkpoint inhibitors used in cancer therapy [29]. The first drug in this class, ipilimumab, was approved for melanoma in 2011 [30]. By releasing constraints on immune cells, these drugs promote immune activation and reinforce anticancer surveillance by modulating immune responses. They not only lead to short-term responses but can also create immune memory, thus allowing for the long-term eradication of tumors and for cures in metastatic cancers previously deemed incurable [31,32]. To this date, one of the best-established biomarkers for response, resistance, and toxicity is the composition of the microbiome.

According to a couple of landmark Science publications, patients with epithelial tumors that responded to immunotherapy had a unique gut microbiome in comparison to non-responders [33,34]. Furthermore, it was shown that fecal microbiome manipulation and transplantation could transform non-responders into responders. We have demonstrated in one of the first-in-human studies that fecal transplant to enrich gut microbiome composition in combination with standard-of-care immunotherapy led to higher response rates than what has been observed in landmark registration trials [35]. Inversely, numerous cohort studies have demonstrated that a reduced diversity of the gut microbiome, often associated with antibiotics use, led to worse survival outcomes in patients receiving immunotherapy [36,37,38]. Interestingly, reduced gut microbiome diversity has also been associated with significant immune toxicities, more specifically immune-related colitis, on checkpoint inhibitors [39]. These toxicities are often refractory to even the strongest immunosuppression strategies and can only be salvaged with fecal transplant [40].

In summary, the human microbiome composition is now recognized as a hallmark of cancer, given its ability to predict responses and toxicities to therapies. Numerous large-scale studies evaluating the impact of microbiome manipulations on cancer outcomes are currently ongoing, and it is expected that many of them will lead to breakthrough novel indications.

## 6. Future Directions

The implementation of precision oncology has resulted in the effective treatment of several deadly cancers by utilizing novel drug delivery mechanisms and by better understanding host factors, such as the microbiome, that modulate anti-tumor responses. Over the past few decades, there has been an overwhelming amount of large-scale biological data generated, primarily due to advances in high-throughput technology. To realize the full promise of precision medicine, further integration of multiple levels of the acquired information is needed. This includes the integration of information at the molecular level (i.e., genome, epigenome, proteome, metabolome), clinical and laboratory data, and environmental factors. A high data analytics capacity and artificial intelligence assistance could be key to this future success [41]. The ultimate goal in the future remains to design specific drugs for each patient’s tumor by leveraging comprehensive multi-omics profiling, including genomics, transcriptomics, proteomics, and immunomics [42].

## Figures and Tables

**Table 1:** A summary of novel and practice-changing oncological treatment approaches.

	Drugs Approved	Mechanism of Action	Novel Class-Specific Toxicity	Oncological Breakthrough
Antibody-drug conjugates (ADCs)	11	Chemotherapy attached to the antibody	Pneumonitis	Allows for delivery of much higher dose of chemotherapy to the tumor than traditionally possible through IV therapy
Bispecific antibodies (BITES)	7	Antibodies targeting 2 receptors at once	Cytokine release syndrome (CRS); immune effector cell-associated neurotoxicity syndrome	Allows one to link the effector immune cells directly to the target antigen of interest in the tumor
Chimeric antigen receptor T-Cell therapy (CAR-T)	6	Genetically modified T cells	Cytokine release syndrome (CRS)	Allows for long lasting remissions using our own immune cells
Microbiome modulation	Nil	Enrichment of flora through stool or prebiotic modulation	Nil	As an adjunct treatment to other anti-cancer therapies, allows for enhanced efficacy and reduced toxicity of such treatments

ADC FDA approvals: Gemtuzumab ozogamicin (multiple myeloma), Brentuximab-veotin and Polatuzumab vedotin and Loncastuximab Tesirine (lymphoma), Trastuzumab emtansine and Trastuzumab deruxtecan and Sacituzumab govitecan (Breast cancer), Inotuzumab ozogamicin (leukemia), Enfortumab vedotin and Tisotumab vedotin and Mirvetuximab soravtansine-gynx (solid tumors); BITE FDA approvals: blinatumomab in acute lymphoblastic leukemia, amivantamab-vmjw in non-small cell lung cancer, tebentafusp-tebn in uveal melanoma, teclistamab-cqyv in multiple myeloma, mosunetuzumab-axgb in follicular lymphoma, epcoritamab-bysp and glofitamab-gxbm in diffuse large B-cell lymphoma; CAR-T FDA indications: idecabtagene vicleucel and ciltacabtagene autoleucel in multiple myeloma, lisocabtagene maraleucel and tisagenlecleucel and axicabtagene ciloleucel in large B-cell lymphoma, brexucabtagene autoleucel in mantle cell lymphoma. Source: https://www.accessdata.fda.gov/scripts/cder/daf/index.cfm (accessed on 18 March 2024).
